# The Optimal Age for Oophorectomy in Women with Benign Conditions: A Narrative Review

**DOI:** 10.3390/jpm15040158

**Published:** 2025-04-19

**Authors:** Aikaterini-Gavriela Giannakaki, Maria-Nektaria Giannakaki, Konstantinos Nikolettos, Christina Pagkaki, Panagiotis Tsikouras

**Affiliations:** 1 First Department of Obstetrics and Gynecology, Alexandra University Hospital, National and Kapodistrian University of Athens, 11528 Athens, Greece; 2 Medical School, Aristotle University of Thessaloniki, 54124 Thessaloniki, Greece; giannakakimarnek@gmail.com; 3 Department of Obstetrics and Gynecology, Democritus University of Thrace, 68100 Alexandroupolis, Greece; k.nikolettos@yahoo.gr (K.N.); christinapagkaki@gmail.com (C.P.); ptsikour@med.duth.gr (P.T.)

**Keywords:** oophorectomy, ovarian removal, benign conditions, ovarian conservation

## Abstract

**Objective:** Oophorectomy is a common procedure for benign uterine conditions, historically recommended for women aged 40–45 and older due to the belief that ovarian preservation had no significant benefits. This review evaluates the literature on the optimal age for oophorectomy in women with benign conditions to assess its risks and benefits and guide clinical decision-making. **Methods:** A narrative review was conducted using a literature search of articles published between January 2000 and February 2025, focusing on the age-related outcomes of ovarian conservation versus removal. **Results:** Oophorectomy remains a complex decision in gynecological surgeries, especially among perimenopausal and postmenopausal women. Evidence supports ovarian conservation in average-risk women, highlighting reduced risks of cardiovascular disease, osteoporosis, and all-cause mortality. Conversely, oophorectomy is favored in high-risk populations, such as BRCA mutation carriers, due to significantly lower risks of ovarian and breast cancers. Despite declining rates, unnecessary oophorectomies persist, influenced by age, socioeconomic status, comorbidities, and surgical approaches. The development of a risk stratification tool offers promise for improving individualized decision-making. **Conclusions:** The decision to perform oophorectomy for benign conditions should be personalized, balancing patient-specific factors to optimize outcomes and long-term health benefits.

## 1. Introduction

Hysterectomy with oophorectomy is one of the most performed major surgeries carried out for benign conditions. Historically, the removal of ovaries was often performed prophylactically in women undergoing hysterectomy for benign conditions, under the assumption that ovarian function beyond reproductive years had limited significance. The prevailing belief was that postmenopausal ovaries no longer contributed to hormonal balance and systemic health, leading to routine oophorectomy at ages 40–45, despite the lack of long-term outcome studies. However, accumulating evidence over the past two decades challenges this traditional approach and has fundamentally shifted this perspective. Research now highlights the critical role of ovarian hormones in preserving cardiovascular health, metabolic function, and cognitive integrity [1]. Salpingo-oophorectomy is a common prophylactic surgery on women with genetic mutations, such as BRCA1 and BRCA2, and a positive family history, as these factors are associated with increased risk of developing ovarian, fallopian tube, and peritoneal cancer. This preventive measure is recommended to significantly reduce the risk of these cancers and improve long-term health outcomes for high-risk individuals [2]. Despite its benefits in cancer prevention, premenopausal oophorectomy leads to hormonal depletion, increasing the likelihood of osteoporosis, dyslipidemia, metabolic syndromes, and cardiovascular disease [3]. The routine practice to remove the ovaries in premenopausal women is still controversial [4], despite its potential to reduce the risk of epithelial ovarian and breast cancers [5].

Given these complexities, the decision-making process for ovarian removal is multifaceted, requiring clinicians to consider age, cancer risk, pre-existing conditions, and hormonal impact to determine whether ovarian conservation or removal is the optimal approach. Recent advancements in individualized risk assessment and personalized treatment algorithms have emphasized the importance of patient-centered strategies rather than standardized protocols [6].

This review critically examines the optimal age for oophorectomy in women with benign conditions, analyzing the risks and benefits associated with ovarian conservation and removal. This study seeks to provide valuable insights to inform evidence-based clinical decisions and enhance understanding of factors influencing oophorectomy.

## 2. Materials and Methods

This review aims to consolidate the existing literature regarding the optimal age for elective oophorectomy in women undergoing hysterectomy for benign gynecologic conditions, examining its long-term effects on cardiovascular health, metabolic stability, and mortality risk.

### 2.1. Search Strategy

This study included articles published between January 2012 and February 2025. A comprehensive search was conducted using PubMed, employing Boolean operators (AND, OR) to refine results. Keywords included ‘ovarian removal’, ‘oophorectomy’, ‘age’, ‘benign uterine conditions’, ‘endometriosis’, ‘adenomyosis’, ‘endometrial hyperplasia’, ‘uterine polyp’, and ‘uterine fibroid’. MeSH terms were incorporated to enhance the precision of search results, ensuring the inclusion of all the relevant literature. References cited in retrieved articles were systematically examined to identify additional studies contributing to the comprehensive analysis of optimal oophorectomy timing in benign uterine conditions. The selection of studies followed predefined eligibility criteria to enhance reliability and minimize bias.

### 2.2. Inclusion Criteria

- Peer-reviewed studies evaluating age-stratified outcomes of oophorectomy and ovarian conservation.
- Randomized controlled trials, retrospective cohort studies, and meta-analyses focusing on cardiovascular, metabolic, and oncological risks associated with ovarian removal.
- Research assessing hormone-replacement therapy (HRT) use post-oophorectomy and its impact on bone density and long-term survival.

### 2.3. Exclusion Criteria

- Studies failing to stratify findings by age, risk level, or HRT usage.
- Case reports, commentaries, and studies with insufficient statistical power or methodological limitations.
- Articles focusing exclusively on malignant ovarian conditions rather than elective oophorectomy for benign indications.

### 2.4. Study Selection

Two independent researchers conducted title and abstract screening, systematically eliminating irrelevant studies to ensure alignment with this review’s objectives. The eligible full-text articles were carefully assessed to select studies addressing the age factor in ovarian removal for benign uterine conditions. The extracted data were critically analyzed, synthesizing key findings into evidence-based clinical recommendations regarding optimal timing for oophorectomy. This review aims to bridge gaps in current knowledge, supporting clinicians in developing individualized surgical strategies that balance cancer prevention and long-term health preservation.

## 3. Results

### 3.1. Overview of Oophorectomy and Health Outcomes

Oophorectomy for benign gynecological conditions presents both advantages and disadvantages, requiring a careful, individualized evaluation to optimize long-term health outcomes. While the procedure eliminates the risk of ovarian cancer, it is associated with several potential risks, particularly when performed at a younger age [7].

- Cardiovascular disease

While ovarian removal eliminates cancer risk, studies highlight increased risks associated with premature oophorectomy (before age 45), including cardiovascular risks, elevated coronary heart disease, and cardiovascular mortality. Women who underwent bilateral oophorectomy before the age of 45 had a 44% higher risk of mortality from cardiovascular disease compared to those who retained their ovaries. However, estrogen treatment through age 45 years or longer helps mitigate cardiovascular risk, but long-term impacts remain debated [4,8].

- Cognitive disorders

When oophorectomy is performed before natural menopause, cognitive decline begins earlier, mimicking age-related deterioration. This means that women who undergo surgical menopause experience cognitive changes at a younger age compared to those who reach menopause naturally [9]. Additionally, oophorectomy before menopause is linked to an increased risk of parkinsonism, likely due to the loss of estrogen, increasing the vulnerability to neurodegenerative diseases [10].

- Bones

Oophorectomy in premenopausal women accelerates bone loss, making fractures more likely, especially in the spine and hip. Low estrogen levels also create conditions favorable for cancer metastases in bones, possibly due to weakened bone integrity and altered cell signaling. Hormone replacement therapy (HRT) partially protects against fractures, reducing incidence by 20%, but additional factors contribute to bone fragility [11].

- Mortality rates

Mortality analysis showed that women undergoing oophorectomy before age 55 have higher mortality rates by age 80 compared to ovarian conservation groups [12]. Excess mortality was recorded at 8.58% for oophorectomy before age 55, decreasing to 3.92% for surgeries before age 59. A lack of HRT significantly increases mortality risk in women under 50. The survival rates stabilize when estrogen therapy is administered, particularly in premenopausal patients [12].

- Quality of Life and Metabolic Consequences

Oophorectomy impacts psychological health, metabolic function, and sexual well-being, particularly in women under 45 [13]. Women aged 45–55 who undergo prophylactic oophorectomy report greater preoperative anxiety and reduced sexual satisfaction [14]. Women undergoing hysterectomy with oophorectomy show higher fat mass and lower lean mass, reinforcing adverse metabolic consequences. HRT mitigates post-oophorectomy complications, reducing hormone-related discomfort and sexual dysfunction [15].

### 3.2. Factors Influencing Oophorectomy Decisions

#### 3.2.1. Age-Based Stratification in Oophorectomy Decisions

##### Oophorectomy Rates by Age

Oophorectomy rates increase with age, particularly after the menopausal transition. A total of 8% of procedures occur in women aged 41–45, 29% occur in women aged 46–50, and 83% are performed in women aged 51+ [16]. Younger patients who undergo bilateral salpingo-oophorectomy (BSO) before 46 experience a 24% faster accumulation of chronic conditions compared to those who retain their ovaries. The first six years post-surgery are marked by a rapid onset of systemic diseases, with stabilization occurring over time [17].

##### Mortality Risk Based on Age at Surgery

The survival data underscore significant age-related differences in mortality following oophorectomy. Women over 45 were significantly more likely to undergo BSO (an odds ratio of 8.421; 95% CI 5.488–12.921). Additionally, ovarian conservation should be prioritized in low-risk premenopausal women, as removing healthy ovaries may lead to long-term health risks, such as cardiovascular disease, osteoporosis, and cognitive decline [18]. Cusimano et al. reported that salpingo-oophorectomy before age 50 is associated with increased all-cause mortality [19,20]. Women under 45 had a 31% higher risk of death (HR 1.31; 95% CI 1.18–1.45; *p* < 0.001), while those aged 45–49 had a 16% higher risk (HR 1.16; 95% CI 1.04–1.30; *p* = 0.007). Women aged 50–54 had a lower risk of death (HR 0.83; 95% CI 0.72–0.97; *p* = 0.018), and those aged 55+ showed no significant association (HR 0.92; 95% CI 0.82–1.03; *p* = 0.16) [19].

##### Impact of Estrogen Therapy on Survival Rates

Oophorectomy performed before age 50 without estrogen therapy is linked to lower survival rates at age 80 compared to hysterectomy alone (52.8% vs. 63.5%). However, when estrogen therapy is introduced, survival rates align closely with those observed in women who underwent BSO after age 50, reinforcing the protective role of estrogen in mitigating post-surgical health risks. On the other hand, women with salpingo-oophorectomy before age 50, but with the use of estrogen therapy, had similar survival rates to those with salpingo-oophorectomy at age 50+, reinforcing the protective role of estrogen [21]. Recent modeling data suggest that oophorectomy in women under 50 with estrogen supplementation does not result in increased mortality risk compared to ovarian conservation. For women under 50, estrogen therapy post-oophorectomy may help mitigate long-term health risks, while in those over 50, the decision to remove ovaries should be personalized, factoring in cancer risk, cardiovascular health, and menopausal symptoms [22]. Age-based oophorectomy decisions are summarized in Table 1.

#### 3.2.2. Low- and High-Risk Populations

##### Ovarian Conservation Versus Elective Oophorectomy

In women without a genetic predisposition to ovarian cancer, ovarian conservation is strongly recommended to reduce cardiovascular disease, osteoporosis, and cognitive decline risk. Studies indicate that prophylactic oophorectomy remains advised for high-risk patients, particularly BRCA mutation carriers. Recent guidelines suggest that elective oophorectomy should be avoided in low-risk women, as it may lead to higher all-cause mortality [23].

##### Stratification of Risk Groups

Other studies concluded that high-risk women with a genetic predisposition (BRCA1/2, Lynch syndrome) may benefit from oophorectomy, while low-risk patients should consider ovarian preservation [18,24,25]. In breast cancer survivors, individualized risk assessments are required before opting for bilateral salpingo-oophorectomy, particularly due to uncertainty in HRT efficacy [26].

##### Surgical Alternatives to Oophorectomy

Ovary-sparing surgery is recommended for benign ovarian masses, preventing unnecessary ovarian removal and long-term health risks. Preoperative risk stratification is essential to distinguish benign versus malignant ovarian lesions, ensuring optimal surgical outcomes [27].

##### Salpingectomy with Delayed Oophorectomy: A Safer Alternative?

Since most epithelial ovarian cancers derive from the fallopian tube, salpingectomy with delayed oophorectomy has gained interest as a potentially safer alternative to immediate ovarian removal. Studies suggest that this approach improves menopause-related quality of life and sexual health, compared to salpingo-oophorectomy. Three ongoing prospective studies—PROTECTOR, SoROCK, and TUBA-WISP II—are investigating the safety and effectiveness of this approach [2].

##### Oophorectomy in Endometriosis Patients

Women with endometriosis who underwent salpingo-oophorectomy showed a lower ovarian cancer incidence compared to endometriosis patients who retained their ovaries. This procedure may serve as a preventive strategy for women with endometriosis and a high-risk profile for ovarian cancer, underscoring the need for better identification of at-risk groups [28]. Salpingo-oophorectomy is a treatment strategy for benign conditions, such as adnexal torsion, tubo-ovarian abscess, ectopic pregnancy, and severe endometriosis [29].

##### Surgeon Preference as a Determinant for Oophorectomy

Surgeon preference remains a key factor influencing whether salpingo-oophorectomy is performed. Studies report higher oophorectomy rates in women aged 45–54, suggesting uncertainty in clinical guidelines for this demographic [30].

##### Chronic Pelvic Pain and Conservative Management Before Hysterectomy

Chronic pelvic pain can stem from various conditions, including endometriosis, uterine fibroids, pelvic inflammatory disease (PID), and adenomyosis. Hysterectomy should remain a last resort, and exhausting conservative treatments are recommended, such as medications, physical therapy, and lifestyle modifications. Careful patient selection for hysterectomy is crucial to prevent unnecessary procedures and optimize surgical decision-making [31]. Oophorectomy management based on risk stratification is summarized in Table 2.

### 3.3. Trends and Concerns over Oophorectomy Practices

Over the past two decades, oophorectomy rates have declined significantly, reflecting growing awareness of the long-term health risks associated with ovarian removal. This shift has been driven by increasing evidence that ovarian conservation offers crucial cardiovascular, metabolic, and cognitive benefits that were previously underestimated [32].

#### Decline in Oophorectomy Rates: Global and Regional Trends

A Taiwanese study reported an 80% decline in oophorectomy rates among women aged 45–49 between 2000 and 2010, reinforcing a more conservative approach in ovarian management [33]. Over the past 14 years (2005–2018), bilateral oophorectomy incidence decreased, particularly among patients undergoing hysterectomy without ovarian indications [34]. In the USA, the annual oophorectomy rates also decreased from 166 per 100,000 women in 2000 to 134 per 100,000 in 2014 [35]. Despite this decline, inappropriate oophorectomy persists, with one-third of premenopausal women undergoing elective ovarian removal without clear medical justification, increasing their risk of cardiovascular disease, osteoporosis, and cognitive decline [36].

### 3.4. Recommendations for Oophorectomy Decisions

Surgical decisions regarding oophorectomy should be guided by integrated risk assessment tools, patient counseling, and evidence-based protocols to ensure ovarian removal is medically justified. Given the long-term health implications of ovarian loss, particularly for cardiovascular, metabolic, and cognitive function, patient-centered approaches should supersede rigid standardized protocols, allowing for individualized surgical strategies based on clinical risk profiles.

To facilitate individualized surgical decision-making, we propose a decision tree framework for clinicians:

- Step 1: Is there a strong indication for oophorectomy? Yes → proceed with surgical removal. No → continue risk evaluation.
- Step 2: Is the patient under 45 without a genetic predisposition? Yes → strong recommendation for ovarian conservation due to cardiovascular and metabolic risks. No → evaluate cancer risk and hormone therapy eligibility.
- Step 3: Is hormone replacement therapy (HRT) required post-oophorectomy? Yes → immediate HRT initiation to prevent premature menopause complications. No → consider alternative hormone management strategies.

## 4. Discussion

### 4.1. Balancing Ovarian Conservation and Elective Oophorectomy

The decision-making process surrounding oophorectomy remains multifaceted, requiring a careful balance between preserving ovarian function and preventing future oncologic risk. While existing evidence strongly supports ovarian conservation in average-risk women, emphasizing its benefits for cardiovascular health, osteoporosis prevention, and overall quality of life, elective oophorectomy remains the optimal choice for high-risk populations, particularly for those carrying BRCA mutations due to their significant risk of ovarian and breast cancer. Recent studies highlight a growing trend in ovarian conservation, especially in women younger than 50, reinforcing the importance of preserving ovarian function to mitigate cardiovascular, metabolic, and cognitive risks. Nevertheless, inappropriate oophorectomies remain in clinical practice, without clear medical indications, necessitating better standardization in clinical practice.

Although patient-specific factors shape surgical decisions, hospital and surgeon variability continue to strongly influence whether oophorectomy is performed, even when evidence suggests ovarian preservation. This underscores the need for standardization in clinical guidelines to ensure evidence-based surgical choices.

Guidelines from the Royal College of Obstetricians and Gynaecologists (RCOG) emphasize the importance of informed consent discussions before hysterectomy for benign conditions. Surgeons must clarify the possibility of unexpected ovarian disease and discuss the circumstances under which oophorectomy may be necessary. A 2013 study by Perera et al. reported that 74% of women under 40 undergoing hysterectomies for benign disease retained their ovaries, reflecting shifting attitudes toward ovarian conservation [37].

### 4.2. Limitations in the Existing Literature

Despite the extensive body of literature on oophorectomy, several critical limitations affect the reliability and generalizability of the findings. A significant portion of the existing evidence relies on retrospective studies, which are prone to bias, limiting the applicability of conclusions to broader populations. Additionally, while tools and scoring systems for risk stratification have been proposed, these often lack prospective validation in diverse populations, raising concerns about their reliability. The full metabolic, cognitive, and psychosocial effects of oophorectomy, particularly in younger women, remain insufficiently studied, leaving knowledge gaps regarding post-surgical hormonal deficits and disease progression risks. Hormone replacement therapy (HRT) has been recognized as a mitigating factor for some adverse effects of oophorectomy, but inconsistencies in data regarding its optimal use and long-term outcomes complicate clinical recommendations.

Addressing these limitations will require large-scale prospective studies and the development of validated risk stratification tools that incorporate genetic, metabolic, and age-based risk assessments. By expanding the evidence base, future research can strengthen clinical guidelines, ensuring they remain both precise and universally applicable.

### 4.3. Future Research Directions

#### 4.3.1. Advancing Evidence-Based Surgical Guidelines

Future research must focus on refining clinical decision-making frameworks, ensuring that surgical recommendations for oophorectomy are guided by validated risk assessment models. Large-scale prospective and randomized clinical studies are needed to strengthen evidence regarding the optimal timing for ovarian removal, particularly in average-risk women, where ovarian conservation may provide long-term health benefits.

#### 4.3.2. Personalized Medicine and Risk-Stratification Tools

The development of standardized risk stratification tools across diverse populations is critical. These tools should integrate age-based risk assessments to determine ovarian conservation suitability, genetic predisposition factors, such as BRCA carriers, hormonal status, and metabolic health indicators, ensuring tailored surgical choices, assessing psychosocial impact metrics, and evaluating long-term effects on mental well-being and sexual health. These tools will enhance individualized surgical strategies, enabling clinicians to make patient-centered recommendations that optimize long-term health outcomes.

#### 4.3.3. Evaluating Hormone-Placement Therapy Strategies

Optimal HRT formulations must be examined across different age brackets to determine their impact on cardiovascular risk, bone density, and cognitive function. Comparative effectiveness studies should evaluate mortality and morbidity outcomes in HRT users vs. non-users, highlighting the clinical benefits and potential risks. Longitudinal studies are necessary to track cognitive, cardiovascular, and metabolic consequences over extended timeframes, ensuring evidence-based HRT protocols.

#### 4.3.4. Exploring Alternative Approaches to Oophorectomy

More research on alternatives to oophorectomy, such as prophylactic salpingectomy with delayed oophorectomy, could expand available options for women at high cancer risk. These approaches have the potential to provide cancer prevention benefits while preserving hormonal and reproductive function. Future studies should evaluate cancer prevention efficacy versus standard BSO approaches, patient satisfaction scores with alternative surgical interventions, and long-term impacts on menopause symptoms, cardiovascular function, and survival rates.

#### 4.3.5. Policy Implementation

Standardized clinical protocols must be established to minimize unnecessary ovarian removals and ensure evidence-based surgical decision-making. Ovarian conservation strategies should be prioritized whenever medically feasible, preventing elective ovarian removal without a clear clinical indication. Advanced training programs for gynecologists should focus on improved risk assessment protocols for elective oophorectomy and regular updates on emerging research findings and evolving surgical best practices.

## 5. Conclusions

This study provides a comprehensive analysis of the factors influencing the decision to perform oophorectomy and its impact on patients’ long-term health and quality of life. It underscores the importance of individualized care and consideration of both the short-term and long-term outcomes on patients’ health. The decision to proceed with oophorectomy should be individualized for each patient.

## Figures and Tables

**Table 1:** Age-based oophorectomy decisions.

Age Group	Outcome	Indication	Reference
41–45 years	8% oophorectomy rate	Ovarian conservation recommended due to higher cardiovascular and metabolic risks.	[16]
46–50 years	29% oophorectomy rate	BSO more likely, but ovarian conservation is beneficial for low-risk women.	[17]
51+ years	83% oophorectomy rate	Higher likelihood of BSO due to cancer risk concerns and menopausal transition.	[18]
<46 years	24% faster accumulation of chronic conditions post-BSO	Early removal leads to higher risks of cardiovascular disease, osteoporosis, and cognitive decline.	[19]
<50 years	Higher mortality risk without estrogen therapy	Estrogen mitigates surgical menopause risks, improving survival rates.	[20]
50–54 years	Lower mortality risk (HR 0.83; *p* = 0.018)	Decision should be personalized, factoring in cancer risk, cardiovascular health, and menopausal symptoms.	[21]
55+ years	No significant difference in mortality risk	Surgical decisions should focus on individualized patient needs.	[22]

**Table 2:** Oophorectomy management based on risk stratification.

Risk Category	Key Findings	Clinical Recommendations	References
Low-Risk Patients (No Genetic Predisposition)	Ovarian conservation is recommended.	Avoid elective oophorectomy unless medically indicated.	[23]
High-Risk Patients (BRCA1/2, Lynch Syndrome)	Prophylactic oophorectomy significantly reduces ovarian cancer risk in genetically predisposed women.	Strong recommendation for oophorectomy in high-risk genetic mutations carriers.	[24]
Women with Breast Cancer History	Individualized risk assessment is necessary before opting for BSO. HRT remains controversial in breast cancer survivors.	Tailored approach required, weighing risks vs. benefits before oophorectomy.	[26]
Benign Ovarian Masses and Low-Risk Indications	Ovary-sparing surgery prioritizes preventing unnecessary removal. Preoperative risk stratification distinguishes benign vs. malignant lesions.	Avoid unnecessary ovarian removal and consider conservative alternatives.	[27]
Salpingectomy vs. Oophorectomy for Cancer Prevention	Most epithelial ovarian cancers originate in the fallopian tube, prompting interest in salpingectomy with delayed oophorectomy.	Delayed oophorectomy offers potentially safer outcomes, improving quality of life.	[2]
Endometriosis and Oophorectomy	Women with endometriosis who underwent BSO showed lower ovarian cancer incidence.	Preventive strategy should be considered for high-risk endometriosis patients.	[28]
Prophylactic Oophorectomy in Premenopausal Women	Rate of elective oophorectomy linked to hospital and physician preferences rather than guidelines.	Standardization needed for surgical decision-making.	[18]
Long-Term Impact on Aging and Health	BSO linked to accelerated aging, including increased chronic conditions within six years post-surgery.	Ovarian preservation should be considered for younger women.	[17]
Practice Variation and Surgeon Preference	Surgeon preference strongly determines oophorectomy rates, particularly for women aged 45–54. Clinical guidelines remain unclear for this age group.	Standardized surgical criteria needed to minimize hospital variation.	[21]
Chronic Pelvic Pain and Hysterectomy	Hysterectomy is a last resort and should only be performed after exhausting conservative treatments.	Careful patient selection ensures unnecessary procedures are minimized.	[31]
Elective Oophorectomy for Benign Gynecological Disorders	Oophorectomy for benign conditions carries long-term risks, including cardiovascular disease, osteoporosis, and cognitive decline.	Ovarian preservation recommended in low-risk patients to reduce post-surgical complications.	[25]
Salpingo-Oophorectomy as Treatment for Benign Conditions	Oophorectomy remains a management strategy for adnexal torsion, tubo-ovarian abscess, ectopic pregnancy, and severe endometriosis.	Clinical guidelines should balance ovarian preservation with surgical necessity.	[29]
Practice Variation in Bilateral Salpingo-Oophorectomy	Surgeon preference is a major determinant of whether BSO is performed, with higher rates among women aged 45–54 years.	Standardized guidelines needed to improve evidence-based surgical decision-making.	[30]

## Data Availability

No new data were created or analyzed in this study.
